# Development of HPLC Method for Quantification of Sinigrin from *Raphanus sativus* Roots and Evaluation of Its Anticancer Potential

**DOI:** 10.3390/molecules25214947

**Published:** 2020-10-26

**Authors:** Anroop B. Nair, Dipal Gandhi, Snehal S. Patel, Mohamed A. Morsy, Bapi Gorain, Mahesh Attimarad, Jigar N. Shah

**Affiliations:** 1 Department of Pharmaceutical Sciences, College of Clinical Pharmacy, King Faisal University, Al-Ahsa 31982, Saudi Arabia; momorsy@kfu.edu.sa (M.A.M.); dmattimarad@gmail.com (M.A.); 2 Department of Pharmacognosy, Institute of Pharmacy, Nirma University, Ahmedabad 382481, Gujarat, India; drdipal.rx@gmail.com; 3 Department of Pharmacology, Institute of Pharmacy, Nirma University, Ahmedabad 382481, Gujarat, India; snehalpharma53@gmail.com; 4 Department of Pharmacology, Faculty of Medicine, Minia University, El-Minia 61511, Egypt; 5 School of Pharmacy, Faculty of Health and Medical Sciences, Taylor’s University, Subang Jaya, Selangor 47500, Malaysia; bapi.gn@gmail.com; 6 Centre for Drug Delivery and Molecular Pharmacology, Faculty of Health and Medical Sciences, Taylor’s University, Subang Jaya, Selangor 47500, Malaysia; 7 Department of Pharmaceutics, Institute of Pharmacy, Nirma University, Ahmedabad 382481, Gujarat, India; jigsh12@gmail.com

**Keywords:** Sinigrin, RP-HPLC, quantification, method validation, cytotoxicity, apoptosis, caspase-3

## Abstract

Sinigrin, a precursor of allyl isothiocyanate, present in the *Raphanus sativus* exhibits diverse biological activities, and has an immense role against cancer proliferation. Therefore, the objective of this study was to quantify the sinigrin in the *R. sativus* roots using developed and validated RP-HPLC method and further evaluated its’ anticancer activity. To achieve the objective, the roots of *R. sativus* were lyophilized to obtain a stable powder, which were extracted and passed through an ion-exchange column to obtain sinigrin-rich fraction. The RP-HPLC method using C18 analytical column was used for chromatographic separation and quantification of sinigrin in the prepared fraction, which was attained using the mobile phase consisting of 20 mM tetrabutylammonium: acetonitrile (80:20%, *v*/*v* at pH 7.0) at a flow rate of 0.5 mL/min. The chromatographic peak for sinigrin was showed at 3.592 min for pure sinigrin, where a good linearity was achieved within the concentration range of 50 to 800 µg/mL (*R*^2^ > 0.99), with an excellent accuracy (−1.37% and −1.29%) and precision (1.43% and 0.94%), for intra and inter-day, respectively. Finally, the MTT assay was performed for the sinigrin-rich fraction using three different human cancer cell lines, viz. prostate cancer (DU-145), colon adenocarcinoma (HCT-15), and melanoma (A-375). The cell-based assays were extended to conduct apoptotic and caspase-3 activities, to determine the mechanism of action of sinigrin in the treatment of cancer. MTT assay showed *IC*_50_ values of 15.88, 21.42, and 24.58 µg/mL for DU-145, HCT-15, and A-375 cell lines, respectively. Increased cellular apoptosis and caspase-3 expression were observed with sinigrin-rich fraction, indicating significant increase in overexpression of caspase-3 in DU-145 cells. In conclusion, a simple, sensitive, fast, and accurate RP-HPLC method was developed for the estimation of sinigrin in the prepared fraction. The data observed here indicate that sinigrin can be beneficial in treating prostate cancer possibly by inducing apoptosis.

## 1. Introduction

Accumulation of active phytoconstituents within the different parts of plants are nowadays explored extensively to use in the ailments of several diseases; therefore, we are re-entering into the era of exploring and using medicinal plants. Researches with fruits, flowers, leaves, seeds, roots, berries, or barks are enormously engaged for identifying the potential chemicals and their exploration as phytomedicine or herbal medicine [1]. Thereby, we encounter tremendous usage of the new buzzword ‘phytochemicals’ in recent research. These studies involved looking for isolated active chemicals within plants instead of using the whole plants as performed by our ancestors. Thus, instead of depending on whole vegetables, fruits, and other food materials, we moved towards supplementation with vitamins and minerals [2]. Various epidemiological studies in the last few decades have demonstrated the importance of consumption of diet rich in fruits and vegetables to reduce the incidence of tumor formation as well as other cardiovascular diseases [3,4,5]. In this perspective, the brassicaceous vegetables are rich in phytochemicals and have demonstrated their nutritional and health benefits [6,7]. Indeed, the vegetables of *Brassica* and *Raphanus* genera are the most widely consumed plants in the Brassicaceae family, which include broccoli, cabbage, brussels sprouts, cauliflower, and Chinese cabbage [8,9]. Concurrently, *Raphanus sativus* L. (radish) is one of the most economically important root crops, cultivated in many tropical countries [10]. The roots and leaves of *Raphanus* are widely accepted and these parts of plants are consumed in the raw as well as cooked form, throughout the world [11]. Roots are reported to contain various phytochemicals—such as phenolics, vitamins, minerals, and glucosinolates—similar to other crucifers [8]. Because of the ingredients present, radish has been explored as a remedial measure in treating various diseases including diabetes, as well as liver and respiratory diseases [10,11,12]. In addition, radish contains some unique bioactive chemical constituents, glucosinolates, a large group of anionic sulfur-containing compounds, which exhibited various biological activities and could be utilized in curing different human diseases [13,14].

Glucosinolates are biologically active plant secondary metabolites which are generally observed in various parts of dicotyledonous angiosperms and are prevalent in *Brassicaceae* family [8,15]. Typically, glucosinolates contain a β-d-thioglucose group linked to a sulfonated aldoxime moiety with a variable side chain derived from amino acids, where its hydrolyzed fraction had shown the potential to prevent carcinogens to the reach and react with the target site or activate the important hepatic enzymes for the protection against several carcinogens [16]. In addition, the literature also demonstrated that the anticancer potential exhibited by the brassicaceous vegetables has a direct relation with their amount of glucosinolate present [17]. Consequently, the quantification of glucosinolate content in the brassicaceous plants is considered to be an essential step in evaluating the anticancer potency of such species. On the other hand, separation and purification of glucosinolates from the vegetables remain a challenging task due to the distinct physicochemical properties they possess [18].

For the analysis of glucosinolates, either the indirect determination of enzyme degradation products or the actual assessment of intact glucosinolates was conducted [19]. In addition, enzymatic or chemically released substances—such as isothiocyanates, oxazolidinethions, thiocyanateion, sulphate, nitrile, or glucose—are also tested in direct analysis. The anticancer potential of various plants of the Brassicaceae family is renowned. Several mechanisms were suggested for the anticancer potential of the cruciferous vegetables including the potential of the phytoconstituents to play as blocking agents against carcinogenesis by quinone reductase activity [7]. Furthermore, glucosinolates and their derivatives can also inhibit the transport of carcinogen molecules to the target site or avoid interaction of sensitive carcinogenic molecules or stimulating the main liver enzymes to safeguard against several carcinogens [16].

**Figure 1:**
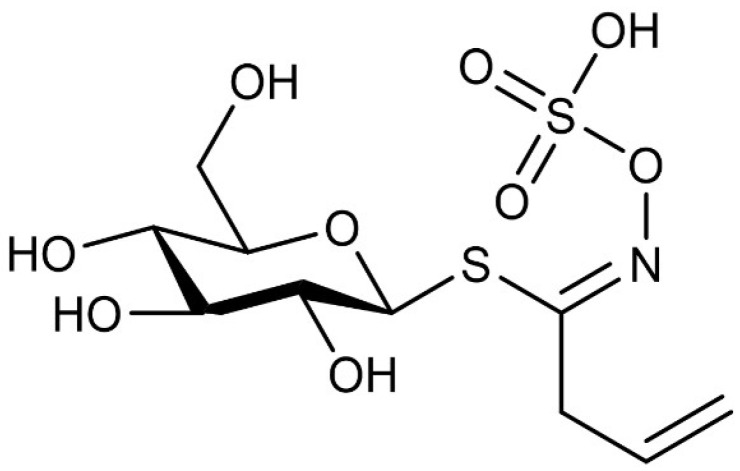
Chemical structure of sinigrin.

The glucosinolates present in radish also hydrolyzes to different bioactive isothiocyanates including sinigrin, sulforaphene, sulforaphane, and indole-3-carbinol [10,20]. Sinigrin (Figure 1), a precursor of allyl isothiocyanate, is chemically (2*S*,3*R*,4*S*,5*S*,6*R*)-3,4,5-trihydroxy-6-(hydroxymethyl)oxan-2-yl] (1*E*)-*N*-sulfooxybut-3-enimidothioate, with a molecular weight of 359.4 g/mol [21]. Sinigrin has bitter taste, though could be masked by various approaches [22,23] and could be used effectively. According to the reports, this molecule is an effective therapeutic agent for the treatment of cancer and inflammation, while established its activity against bacteria and fungi and also possesses antioxidant, and wound healing effects [18,24].

Development of an analytical method to estimate sinigrin in the radish roots is of utmost importance to quantify the content. Few methods had been explored to quantify various glucosinolates available in brassicaceous leaf, seed, and root [25,26]. Similarly, quantification of sinigrin from mustard [27] and other Brassica vegetables [28,29] had also been reported. However, these methods include tedious extraction procedures or the use of sophisticated instruments. On the other hand, there is no report available on a simple reverse phase-high performance liquid chromatography (RP-HPLC) method to quantify sinigrin in radish. Thus, the objective of this study was to develop and validate a simple RP-HPLC method to quantify the sinigrin content in the *R. sativus* roots and to evaluate the anticancer potential of the sinigrin-rich fraction by performing cell line studies. The study also evaluates to establish a possible molecular mechanism of sinigrin in the treatment of cancer.

## 2. Results 

### 2.1. Quantification of Sinigrin Using RP-HPLC Method

**Figure 2:**
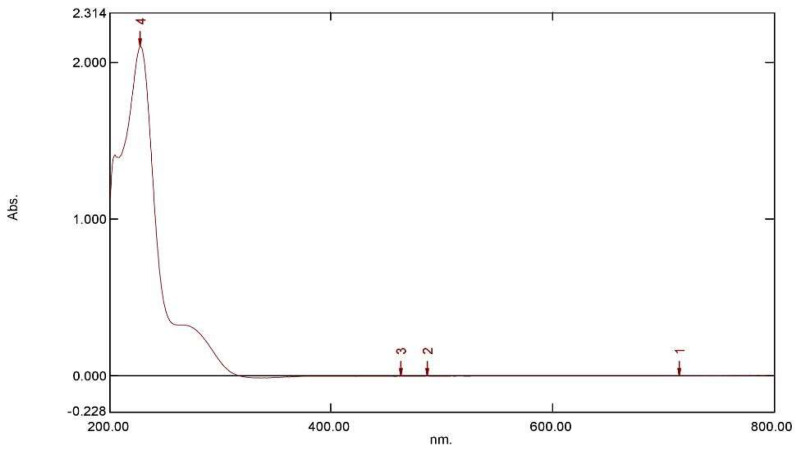
UV–visible absorption spectra of sinigrin in 0.02 M TBA in water (pH 7): ACN (80:20%, *v*/*v*).

A UV spectral scanning for sinigrin was evaluated using 0.02 M tetrabutylammonium (TBA) in water (pH 7): acetonitrile (ACN) (80:20%, *v*/*v*). The maximum UV absorption was found to be at 227 nm wavelength (Figure 2). Therefore, 227 nm was selected for sinigrin detection in the present study.

**Figure 3:**
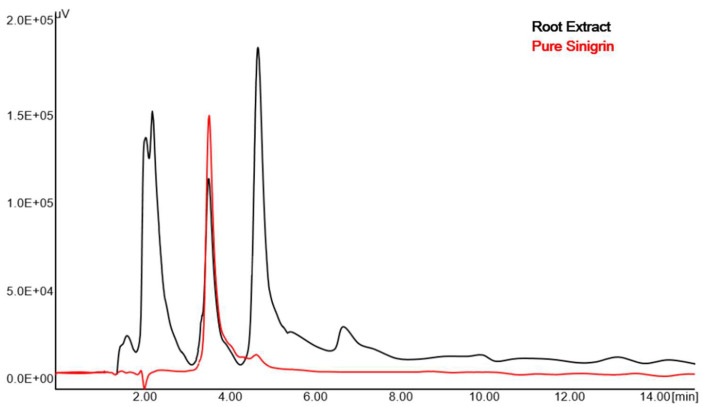
Overlay of chromatogram of prepared fraction of *Raphanus sativus* roots and pure sinigrin at 227 nm.

The chromatogram of sinigrin showed a single peak at retention time of 3.592 min, which is the most appropriate for the assay determination. At optimized chromatographic conditions, prepared sinigrin-rich fraction showed a well-separated peak of sinigrin. Figure 3 represents the RP-HPLC chromatograms of sinigrin in the prepared fractions using the *R. sativus* roots at retention time of 3.608 min using PDA detector. The amount of sinigrin in prepared fraction obtained using the RP-HPLC method is 0.59% *w*/*w*.

### 2.2. Validation of RP-HPLC Method

#### 2.2.1. System Suitability

The verification of reproducibility of the HPLC system is generally carried out using system suitability testing. The values obtained in different parameters evaluated are good (Table 1), which confirm that the performance of the HPLC system was appropriate for the analysis of sinigrin.

#### 2.2.2. Linearity and Range

A six-point calibration curve was established by analyzing different concentrations of sinigrin and the peak area was determined for each concentration. The linearity curve was achieved by plotting the observed chromatogram peak areas versus concentrations (50–800 µg/mL). The correlation coefficients (R^2^ values) are summarized in Table 1.

#### 2.2.3. Limit of Detection and Limit of Quantification

As per the International Council on Harmonization (ICH) guidelines the sensitivity of the developed method is determined by calculating the limit of detection (LOD) and limit of quantification (LOQ). The low LOD and LOQ results summarized in Table 1 showed that the developed method was sensitive.

#### 2.2.4. Specificity

The comparison of pure sinigrin chromatogram with that of roots extract (Figure 3) showed that the extract constituents did not interfere with the peak of sinigrin. Furthermore, the sinigrin from the fraction was eluted at the same retention time of pure sinigrin, signifying the specificity of the method.

#### 2.2.5. Accuracy and Precision

The accuracy and precision data of sinigrin were mentioned in Table 2. The average %RE obtained for accuracy of the proposed method was found to be −1.37% and −1.29%, while the precision (percentage relative standard deviation (%RSD)) of the developed method was 1.43% and 0.94% for inter-day and intraday, respectively (Table 2). Obtained results are indicative on accuracy and precision of the developed method within the analytical range.

#### 2.2.6. Recovery Study

The mean recovery of sinigrin from the ethanol extract was found to 95.66% with a low %RE (−4.54%) indicating the negligible matrix effect on the recovery (Table 3).

#### 2.2.7. Stability

The analysis results of benchtop and refrigerated extract showed less than 2% RSD, indicating the stability of sinigrin in the extract for more than 24 h at room temperature and more than 7 days within the refrigerator at 4–8 °C.

### 2.3. Anticancer Potential of Sinigrin on Different Cell Lines

The data observed in the current study signifies that the amount of sinigrin present in the prepared fraction and *IC*_50_ values are interrelated. Indeed, the half-maximal inhibitory concentration (*IC*_50_) value was found to decrease when the quantity of sinigrin in the prepared fraction increased. It is well-known that the lower the *IC*_50_ value, higher is the potency of the compound against cell line. The *IC*_50_ values of roots fraction, sinigrin, and colchicine on DU-145, HCT-15, and A-375 cell lines are summarized in Table 4. Higher potency of roots fraction was noticed with the DU-145 cell line as compared to other cell lines evaluated (Table 4). In addition, the *IC*_50_ value of colchicine (11.92 µg/mL) in the DU-145 cell line was comparable with the pure sinigrin (10.91 µg/mL) and prepared fraction (15.88 µg/mL), which substantiates the anticancer potential of the roots fraction and pure sinigrin (Table 4). Moreover, it can be observed from the findings that the prepared sinigrin-rich *R. sativus* root extract exhibited highest inhibitory effect against the DU-145 cell line; thus, this cell line was used for the further apoptotic and caspase assay.

### 2.4. Effect of Roots Extract of Raphanus sativus and Sinigrin on Apoptotic Assay

**Figure 4:**
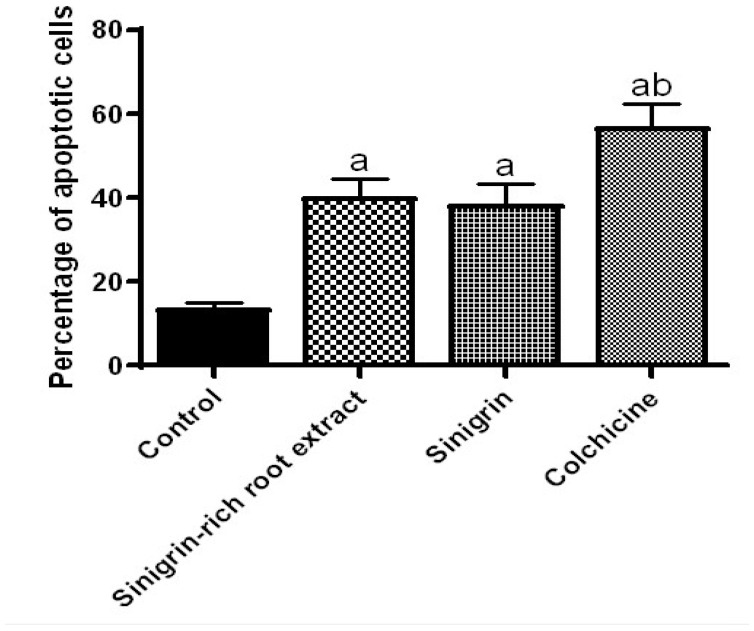
Effect of various treatments on apoptosis using flow cytometry assay using annexin V-fluorescein-5-isothiocyanate staining. DU-145 prostate cancer cells were treated with the medium in control cells. DU-145 cells were exposed to 25 μg/mL of the sinigrin-rich root extract, sinigrin, and colchicine for 24 h and were subjected to further evaluation of cell apoptosis by flow cytometry. Values were represented as the mean ± SD. “a” Significant (*p* < 0.01) difference from the control group and “b” significant (*p* < 0.05) difference from the sinigrin group.

Results of flow cytometry have positively shown that the treatment with *R. sativus* roots extract, sinigrin, and colchicine at concentrations of 25 µg/mL for 24 h, there is considerable increase in cell apoptosis percentage when compared to the control in all treatment groups (Figure 4). The results of this study indicated that the tested samples induce apoptosis to the experimental cell line.

### 2.5. Effect of Roots Extract of Raphanus sativus and Sinigrin on Caspase-3 Activity

**Figure 5:**
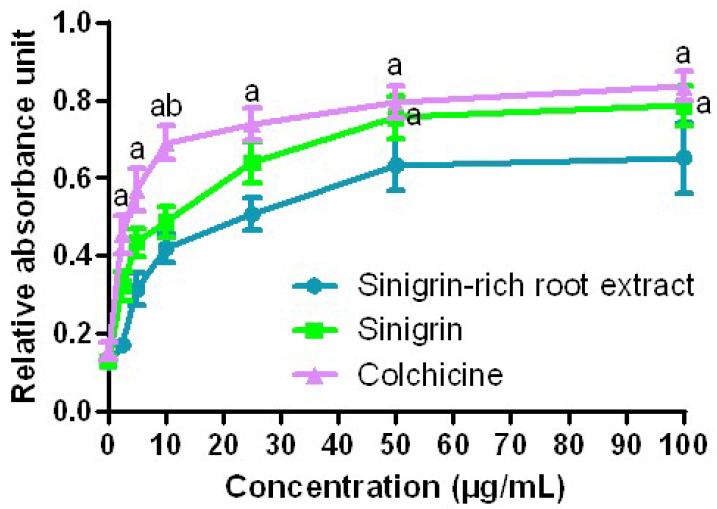
Effect of various concentrations (0, 2.5, 5, 10, 25, 50, and 100 μg/mL) of sinigrin-rich root extract, sinigrin, and colchicine on activities of caspase-3 in the DU-145 prostate cancer cells for 24 h. Values are represented as the mean ± SD (*n* = 3). “a” Significant (*p* < 0.01) difference from the sinigrin-rich root extract group and “b” significant (*p* < 0.05) difference from the sinigrin group.

Activation of caspase is a usual mechanism whereby chemotherapeutic compounds cause apoptosis. Therefore, the degree of caspase-3 activation was assessed to get more insight into the mechanism of action. In general, various chemotherapeutic agents ultimately follow a common apoptotic trail mediated by the activation of caspases (cysteine-dependent aspartate-specific proteases). In order to examine the participation of caspases in *R. sativus* roots extract and sinigrin-mediated apoptosis, activity of caspase-3 in apoptotic process was studied in DU-145 cells upon incubation with *R. sativus* roots extract, sinigrin, and colchicine at different concentrations for 24 h. In our study, the observed assay values signify that the activities of caspase-3 in the *R. sativus* roots extract and sinigrin treated DU-145 cells are increased substantially in a dose-dependent manner (Figure 5).

## 3. Discussion

Sinigrin is one of the active phytoconstituents present in *R. sativus*, which is known to produce pharmacological effects. This activity can be directly related to the phytochemical content; therefore, the determination of the amount of sinigrin in the roots remains an essential step in examining the anticancer potential. However, the separation of sinigrin from the *R. sativus* roots is a difficult task due to the presence of the sulfate group and the thioglucose moiety [18]. Moreover, the roots of *R. sativus* contain about 90% of the water content and the bioactive phytoconstituents get degraded because of enzyme myrosinase. Therefore, a stable freeze-dried powder of radish root was prepared initially and then isolation of sinigrin was carried out with multiple steps to remove other components. In the current study, an ion exchange chromatography was used to separate sinigrin, a polar compound, from other plant components. The prepared fraction was further used for quantifying the content of sinigrin using the RP-HPLC method. 

Separation and resolution of the HPLC method are mostly affected by different elements such as column, detector, mobile phase, etc. [30,31]. Different analytical columns (C8/C18; Intersil/Zorbex; 250 × 4.6 or 150 × 4.6), mobile phase (water, acetonitrile, buffer) with different ratios and varying flow rates (0.5 or 1 mL) at the detection wavelength of 227 nm was assessed to optimize the HPLC conditions. From the different trials, a column with the C18 stationary phase was selected which gave proper retention, good theoretical plates, and resolution between extract and pure sinigrin.

It is reported that the TBA could be used as an ion-pairing agent to separate polar compounds [32]. Hence, TBA was selected in the current study and included in the mobile phase for estimation of sinigrin. The ratio and concentration of the mobile phase were varied to achieve a good chromatogram as well as a short retention time, without compromising the selectivity of the method. From the different applied mobile phases, the mobile phase consisting of 0.02 (M) TBA:ACN (80:20, *v*/*v*) was found to be satisfactory. HPLC chromatogram of sinigrin in the prepared fraction showed good separation with better resolution of the peak by isocratic elution of the selected mobile phase using UV absorbance at λ_max_ 227 nm. Indeed, the HPLC chromatogram of sinigrin in the prepared fraction was comparable with the standard solution of the pure drug.

The specificity describes the potential of the developed method to separate the active constituent from the remaining components. Therefore, the absence of interfering peaks of other components in the chromatogram could be considered specific. Indeed, the chromatogram of *R. sativus* roots extract was similar to the pure drug with a similar retention time (3.6 min) without any interferences from the extract components. In applied chromatographic conditions, there was no merging of other peaks with sinigrin, thus the developed method is specific for the estimation of sinigrin in radish. In the current study, the developed analytical method for determining sinigrin from the root extracts of *Raphanus sativus* showed the retention time of sinigrin as 3.6 min at a flow rate of 0.5 mL/min. Comparing the retention time of published methods for sinigrin quantitation from tomato leaves by Mvumi and team, it is 7.217 min [33]. The results of the mentioned research referred to a quite long run time that needs to be performed for the determination of sinigrin, which is a loss of time and solvent. Alternative researches reported 3.7 min retention time of sinigrin while quantified from the mustard seed at a flow rate of 1 mL/min [27], and 2.8 min at a flow rate of 1 mL/min [34], where the retention time is equivalent to this research; however, the increased flow rate signifies high consumption of solvent during analysis. Therefore, it could be said that our method is economical and thus, novel compared to the established methods.

The calibration curve of sinigrin indicates perfect linearity and high regression coefficient. In addition, the linearity range observed in this study signifies that sinigrin in a wide range could be assessed, which is an additional advantage of the developed method. Moreover, in the case of sinigrin, the therapeutic effect is also related to its content; therefore, one can also corroborate the sinigrin level to the test result [35].

The accuracy and precision of the proposed method describe the closeness between the experimental and actual values [36]. The observed inter-day and intraday accuracy and precision data signify the accuracy and precision of the developed method, where the precision values are <2% for the repeated analysis of the set of quality control samples, which meet the standard criteria of ICH harmonized tripartite guideline [37].

The excellent recovery of added sinigrin from the ethanol extract with very low %RE showed low interference of matrix on the extraction procedure. Further, our data on recovery is in accordance with the literature [38]. However, our results on recovery are even better than the recent report by Yuan and team [39], where they reported that the average recovery of 88.81 ± 4.41%. The promising stability data observed with the developed method also implies the stability of sinigrin in the extract for more than a day at room temperature and a week in refrigerated condition. Overall, the developed HPLC method was established as satisfactory, with the desired accuracy, and precision for sinigrin separation and quantification from the root extract without the interference of other phytoconstituents.

Cytotoxicity of drug molecules can be predicted using cell lines, in absence of animals [40,41]. In the current study, three cell lines were selected to evaluate whether the prepared sinigrin-rich *R. sativus* roots extract has similar anticancer potential as observed with pure sinigrin. The cytotoxic potential of prepared sinigrin fraction and pure sinigrin was compared with colchicine, another natural compound with approved cytotoxic activity [42,43], and the *IC*_50_ values were compared. The results of the anticancer activity of sinigrin-rich *R. sativus* roots extract showed excellent inhibitory activity against three different cell lines. Previous studies showed the anticancer potential of sinigrin in an animal model of hepatocellular carcinoma as well as in bladder cancer [44]. Our results showed that *R. sativus* roots extract and sinigrin showed a cytotoxic effect against all three cell lines and restrained the growth of the cells in a dose-dependent manner. In addition, according to the result of the 3-(4,5-dimethylthiazol-2-yl)-2,5-diphenyltetrazolium bromide (MTT) assay it was observed that both roots extract and pure sinigrin possess the highest anticancer potential in the DU-145 cell line. Thus, it can be hypothesized that sinigrin or sinigrin containing extracts might have significant beneficial effects on prostate cancer. On the other hand, the *IC_50_* values of sinigrin and sinigrin-rich *R. sativus* root extracts in HCT-15, and A-375 cell lines were found different. This difference in *IC*_50_ values of sinigrin and sinigrin-rich *R. sativus* root extracts might be explained by the presence of other constituents within the sinigrin-rich *R. sativus* root extract, whereas sinigrin is used as pure. The lower content of sinigrin might result in slightly higher *IC*_50_ values against the tested cancer cell lines when treated with sinigrin-rich *R. sativus* root extract.

Apoptosis is an orderly process of genetically programmed cell death and is mediated by the action of genes and proteins that activate caspases pathways that do not provoke an inflammatory response. It is also documented that the extent of apoptosis influence anticancer activity. Therefore, induction of apoptosis can be a target of many anticancer agents [45]. Previous reports revealed that sinigrin produced apoptosis in the liver cancer cell line through downregulation of caspases, signifying its potential as an anticancer agent against liver cancer [44]. Similarly, sinigrin demonstrated good inhibition of bladder cancer growth when evaluated in an orthotopic rat model [46]. In the current study, flow cytometry was employed to assess whether *R. sativus* roots extract could produce cell death in DU-145 cells through apoptosis induction. The data demonstrated that the *R. sativus* root extract and sinigrin have anticancer potential which is mainly mediated by induction of apoptosis. Among all caspases, caspase-3 is very essential for the activation of apoptosis by catalyzing the majority of caspase substrates, which contributes to the functional consequences associated with apoptosis [47]. The results observed in the caspase assay signifies that the activation of caspases-3 augmented by treatment with *R. sativus* roots extract and sinigrin dose-dependently. Thus, the results of the current study on caspase-3 activity further confirmed the hypothesis that *R. sativus* and sinigrin showed anticancer activity by induction of apoptosis through the activation of caspase-3. 

## 4. Materials and Methods

### 4.1. Chemicals and Reagents

Sinigrin hydrate, TBA hydrogen sulfate, sodium acetate, potassium sulfate, and DEAE-Sephadex A-25 were procured from Sigma-Aldrich (St. Louis, MO, USA). Enzyme sulfatase (Helix pomatia Type H-1), Dulbecco’s modified Eagle’s medium (DMEM), trypsin, ethylenediaminetetraacetic acid, penicillin-streptomycin solution, fetal bovine serum, trypan blue, MTT, and Annexin V-FITC apoptosis detection kit were obtained from Thermo Fisher Scientific (Waltham, MA, USA). Caspase-3 activity assay kit was obtained from Elabscience (Houston, TX, USA). Human prostate cancer cell line (DU-145), human colon adenocarcinoma cell line (HCT-15), and human melanoma cell line (A-375) were generously provided by the National Centre for Cell Science (Pune, Maharashtra, India). All the cells were grown in DMEM culture media. HPLC grade methanol, water, and ACN were purchased from SD Fine-Chem (Mumbai, Maharashtra, India).

### 4.2. Collection of Plant Specimen

Fresh good quality of *R. sativus* roots was collected from the local market (Ahmedabad, Gujarat, India). The obtained roots were cut into small pieces and crushed using a mixer grinder to get semisolid mass. This product was then kept in a flask used for freeze-drying and lyophilized for 4 days to obtain a completely dried powder, which was used for further investigation. The freeze-dried product was kept in the refrigerator in an airtight container until further use.

### 4.3. Preparation of Sample Extracts

A 50 g quantity of the freeze-dried powder of *R. sativus* roots was extracted with 250 mL of ethanol 70% (*v*/*v*) for 15 min at 65 °C and centrifuged. The supernatant was removed and the left marc was extracted repeatedly twice with the same amount of 70% ethanol and centrifuged again. Thereafter, the extracts were drained and combined to get the fraction containing glucosinolate. For removal of inorganic impurities in crude extracts, the ethanol was evaporated, and approximately 2.5 g dried residue was obtained. The residue obtained was further taken for the extraction of glucosinolates using ion exchange resins [48]. The filtrate having glucosinolate fraction was allowed to pass through DEAE-Sephadex A-25 resin column along with acetate buffer to adjust acidic pH near 5.5. After loading the content, the resin column was further washed with buffer and mixture of a solution of formic acid:2-propanol:water with a ratio of 3:2:5. Then the column was eluted with potassium sulphate solution prepared in water. The collected extract was reduced and further purified by adding absolute ethanol, and evaporated to obtain the residue. Purification was done using sulfatase (from Helix pomatia type H-1) after the addition of sodium acetate. Furthermore, the filtrates were filtered using a Millipore immersion filter (0.45 µm) and used for chromatographic HPLC analysis.

### 4.4. Chromatographic Conditions

The quantitative analysis of sinigrin was performed using a Jasco HPLC system (2000 series, Jasco, Tokyo, Japan) fitted with a pump (Jasco PU-2080 Plus), mixer (Jasco MX-2080-31), single loading injector (Rheodyne Model 7125 with 20 μL fixed loop), and PDA detector (Jasco 2075 Plus). The chromatographic evaluation was conducted using Jasco Borwin software, version 1.50. The chromatographic separation was achieved using C18 analytical column (Inertsil; 150 × 4.6 mm internal diameter, 5 μm particle size) set at room temperature (25 ± 2 °C) with isocratic mobile phase consisting of 0.02 M TBA in water (pH 7): ACN (80:20%, *v*/*v*) at a flow rate of 0.5 mL/min with an injection volume of 20 μL.

### 4.5. Preparation of Stock Solutions

The required amount of sinigrin was precisely weighed and dissolved in HPLC grade water at 1000 µg/mL concentration to get the stock solutions. Serial dilution of the primary stock solutions was made in water to get working standard solutions (50, 100, 200, 400, 600, 800 μg/mL). These freshly prepared solutions were used as a working standard solution for the preparation of the calibration curve and further chromatographic analysis.

### 4.6. Validation of Developed RP-HPLC Method

The optimized RP-HPLC method for sinigrin was validated as per ICH Tripartite guidelines Q2 for system suitability, linearity and range, LOD, LOQ, specificity, accuracy, precision, recovery, and stability [37,49].

#### 4.6.1. System Suitability

System suitability testing is essential to check and confirm the functioning as well as the reproducibility of the HPLC system during analysis [50]. Briefly, the standard solution (400 μg/mL) of sinigrin was injected in triplicate and various variables including retention time, peak area, theoretical plates, and asymmetry of sinigrin were measured.

#### 4.6.2. Linearity and Range

The linearity of sinigrin in various drug concentrations (50–800 μg/mL) was determined by plotting a standard curve with peak area against the concentration at six levels. The samples were injected thrice and the peak area obtained in each chromatogram was measured, and the calibration curve was prepared. The solutions for assessing the linearity of sinigrin were prepared as described above in the preparation of stock solutions. A regression equation was used to determine the sinigrin content in the prepared extract.

#### 4.6.3. LOD and LOQ

LOD and LOQ were estimated according to the standard equation, using the standard deviation (SD) data noticed at very low concentration as well as the slope observed. The equations used are; LOD = 3.3 α/S and LOQ = 10 α/S, where α is the SD of the signal at very low concentration and S is the average of the slope of three linearity curves [51].

#### 4.6.4. Specificity

Determination of specificity of the analytical method was performed by assessing the chromatogram of blank (diluents and mobile phase) in the absence of drug. Furthermore, the standard sinigrin solution was analyzed to check the possible interference due to blank or other peaks in prepared plant extracts.

#### 4.6.5. Accuracy and Precision

The intraday and inter-day accuracies of the proposed HPLC method was carried out by standard addition method. Different concentrations of sinigrin were added (50, 400, and 800 μg/mL) to the predetermined amount and the quantification was done by the developed method. Similarly, the intraday precision was determined by injecting the three different concentrations (50, 400, and 800 μg/mL) of sinigrin three times on the same day. Inter-day precision was determined by injecting the same solutions for three days. The precision of the proposed method was shown as a %RSD and accuracy was confirmed by percent relative error [52]. 

### 4.7. Recovery Study

Recovery study was performed by applying the standard addition method by adding the known amount of standard sinigrin to the ethanol extract. Furthermore, the extract was subjected to the purification process and analyzed by the proposed HPLC method. The study was done at 50%, 100%, and 150% of the concentration levels in triplicates. Recovery study was express as percent recovery and percent relative error.

### 4.8. Stability Study

For determining the stability of sinigrin in roots extract, the sample was evaluated at different handling and storage conditions such as benchtop and refrigerator. For benchtop stability, the extract was analyzed and stored for 24 h at room temperature. In addition, the extract was stored in the refrigerator for 7 days and then analyzed.

### 4.9. Cell Lines and Culture

The prepared sinigrin-rich *R. sativus* roots fraction was evaluated against DU-145, HCT-15, and A-375 cell lines. The effect of prepared sinigrin-rich fraction and standard sinigrin were compared with colchicine and the *IC*_50_ values were calculated. All the media used were accompanied by 10% fetal bovine serum and an antibiotic combination containing penicillin (5 mg/mL) and streptomycin (5 mg/mL). The exponentially growing cultured cells were further used for experiments.

### 4.10. Screening of Test Compound by MTT Assay

The desired final concentration to seed the cells was kept at 5 × 10^4^ cells/well, which was exposed to 100 µL culture medium and a single microplate (tissue culture grade, 96 wells, flat bottom) was treated with the final concentration of the test compound (333–0.05 μg/mL). The incubation of cell cultures was done for 24 h at 37 °C, 6.5% CO_2_, and 75% relative humidity following the established method [53]. 10 µL MTT labeling mixture was added and each well was diluted to 100 µL with solubilization solution and the prepared mixture was incubated for 4 h. The formazan product was measured by recording the absorbance at 580 nm using a microplate ELISA reader. The evaluation of the data was done by measuring absorbance and the corresponding chemical concentrations were calculated. Dose response curve is explained by linear regression analysis by keeping 95% confidence limit along with R^2^ and the *IC*_50_ was calculated. Percentage cytotoxicity was determined the using below Equation (1)

(1) $$\%\;viability\;=\;\frac{\left(A_{T}-A_{B}\right)}{\left(A_{C}-A_{B}\right)}\times 100$$

where *A_T_* = Absorbance of treated (compound), *A_B_* = Absorbance of blank (only media), and *A_C_* = Absorbance of control (untreated). 

Thus, Cytotoxicity = 100 − % cell survival.

GraphPad Prism software version 5 (San Diego, CA, USA) was used to derive the *IC*_50_ value using a curve fitting method. The values of log concentration of drug and percentage inhibition of cell growth or % cytotoxicity were considered and the graph was plotted by keeping concentration on X-axis and % cytotoxicity on the Y-axis. By taking the drug concentration at a 50% position on the Y-axis, *IC*_50_ was estimated. The relationship was shown by constructing the sigmoidal curve.

### 4.11. Detection of Apoptosis by Flow Cytometry Assay

DU-145 cells were used for carrying out the in vitro apoptosis assay. Adequate cell number was sown into 6 well plates and allowed to adhere to growth media. After 24 h, a confluent monolayer was achieved which was separated into various groups; dimethyl sulfoxide control, sinigrin-treated, and colchicine-treated. The respective concentrations of the tested compounds were included in the confluent monolayer in the complete growth media and plates were incubated for 24 h, according to the regular MTT protocol. Cells were then washed thrice using phosphate-buffered saline, and later digested and centrifuged gradually. The detection of apoptotic cells was made by flow cytometry (Thermo Fisher Scientific) after staining with an Annexin V-FITC [54].

### 4.12. Measurement of Caspase-3 Activity

To assess the caspase activity, the cultivated cells (1 × 10^6^) in 25 cm^2^ flasks were processed with 2.5, 5, 10, 25, 50, and 100 μg/mL of the sinigrin-rich root extract, sinigrin, and colchicine for 24 h. The working of caspase-3 was measured with the help of caspase-3 colorimetric assay kit and the protocol of the manufacturer was followed [55].

## 5. Conclusions

A simple and sensitive HPLC method has been developed and validated for quantification of sinigrin, which could be utilized to precisely estimate sinigrin content in *R. sativus* roots extract without the interference of other phytoconstituents. The MTT assay results demonstrated the potential cytotoxic activity of sinigrin in the prostate cancer cell line (DU-145), while the apoptotic and caspase-3 activity assays suggest the activity could be probably by inducing apoptosis. Indeed, the data observed here demonstrated that *R. sativus* roots have a rich source of sinigrin and could be utilized as one of the potent remedies against cancer or associated ailments. In a nutshell, *R. sativus* roots are plentiful around the world, and they have demonstrated the anticancer effects; hence could be useful to further investigate its activity on other cancer models of animals and humans. However, our findings may be expanded to evaluate in different animal models for effective and safe control against this life-threatening disease.

## Figures and Tables

**Table 1:** System suitability and validation parameters for sinigrin (400 ppm).

Parameters	Results
Retention time ± SD (min)	3.59 ± 0.12
Peak Area ± SD	6,451,361 ± 20,673
Theoretical plates ± SD	4005.29 ± 42.56
Symmetry ± SD	1.39 ± 0.08
Linearity range (μg/mL)	50–800
Slope ± SD	15,005 ± 154
Intercept ± SD	467,329 ± 2475
Correlation coefficient (R^2^)	0.999
Limit of detection (μg/mL)	0.78
Limit of quantification (μg/mL)	2.45

**Table 2:** Results of accuracy and precision data of sinigrin.

Analyte	Amount of Sinigrin Added (µg/mL)	Intraday			Interday		
		Amount Detected Mean ± SD ^a^	%RSD	%RE	Amount Detected Mean ± SD ^b^	%RSD	%RE
Sinigrin	50	49.45 ± 0.87	1.76	−1.11	49.45 ± 0.49	0.99	−1.11
	400	392.82 ± 5.98	1.53	−1.82	394.54 ± 4.56	1.16	−1.38
	800	790.55 ± 7.89	1.01	−1.19	14 ± 5.02	0.64	−1.37
	Average		1.43	−1.37	-	0.94	−1.29

^a^*n* = 3; ^b^ *n* = 9; RSD: Relative standard deviation; RE: Relative error.

**Table 3:** Recovery study data.

Analyte	Level (%)	Amount of Sinigrin (µg /mL)	Amount Detected (Mean ± SD)	% Recovery	% RE
Sinigrin	50	200	189.45 ± 2.84	94.73	−5.57
	100	300	289.82 ± 3.42	96.61	−3.51
	150	400	382.55 ± 4.79	95.64	−4.56
	Mean			95.66	−4.54

**Table 4:** *IC*_50_ values of sinigrin and prepared *R. sativus* roots fractions of on DU-145, HCT-15, and A-375 cell lines.

Sample	*IC*_50_ Values (µg/mL)		
	DU-145	HCT-15	A-375
Colchicine	11.92	12.17	10.17
Sinigrin	10.91	16.76	7.37
Sinigrin-rich root extract	15.88	21.42	24.58
